# Efficacy of a Culturally and Linguistically Competent Community Health Coach Intervention for Chinese with Hypertension

**DOI:** 10.31372/20200503.1087

**Published:** 2020

**Authors:** Wen-Wen Li, Donna Lew, Linda Quach

**Affiliations:** a San Francisco State University, United States; b American Heart Association, United States

**Keywords:** health coach, hypertension, medication adherence, Chinese immigrants

## Abstract

**Purpose:** To develop and pilot test the efficacy of a culturally and linguistically sensitive, community health coach (CHC)-based intervention in Chinese immigrants in improving blood pressure control and medication adherence.

**Design:** This study was conducted in 2017 with a cross-sectional design (*n* = 23). A CHC intervention was implemented using one 25-minute group educational presentation plus one 10-minute question and answer session at baseline, followed by four, 10-minute bi-weekly group question-and-answer sessions.

**Findings:** There was a significant reduction in both systolic and diastolic blood pressure from baseline to week 8: Systolic BP −17.33 (±11.32) (*p* < 0.005) and diastolic BP −9.58 (±6.57) (*p* < 0.005). The mean score for medication adherence was 10.56 (±3.24) (possible range 3–15) at baseline and there was no significant change at week 8 (mean 10.89 ± 3.95) (*p* = 0.86).

**Conclusion:** The CHC-based hypertension management program showed significant reductions in both systolic and diastolic blood pressures in Chinese immigrants. Since the proposed CHC-based hypertension management program is low cost and easy to establish, further investigation is recommended to generate more results for comparison.

**Practice Implications:** There is potential for the CHC intervention to be implemented in clinical settings to help Chinese immigrants at large achieve optimal blood pressure control.

## Introduction

Blood pressure control (BPC) reduces the risk of developing complications of hypertension (HTN), such as cardiovascular disease (Bloch & Basile, 2019) and stroke, thereby lowering rates of early morbidity and mortality (Go et al., 2013). Among the 25 million U.S. adults who lack BPC (Go et al., 2013), Chinese immigrants are at particular risk (Yi, Thorpe, Zanowiak, Trinh-Shevrin, & Islam, 2016) as HTN disproportionately affects Asian Americans and their counterparts in Chinese countries compared to non-Hispanic whites (Curb, 2010). Chinese people not only constitute the largest (23%) Asian group in the U.S. (Ngo-Metzger et al., 2003; U.S. Census Bureau, 2010), but also have disproportionately high rates of uncontrolled HTN due to many factors with a major one being the lack of a culturally specific HTN management intervention (Duong, Bohannon, & Ross, 2001; Li, Stewart, Stotts, & Froelicher, 2006; Li, Wallhagen, & Froelicher, 2008). However, only half of Chinese immigrants had their blood pressure controlled (Chen & Hu, 2014). Considering that Asians are projected to be the fastest growing immigrant group in the U.S. (38% of immigrants by 2055) (Radford, 2019), addressing HTN is an important step towards tackling the country’s health disparities.

Chinese immigrants differ from other U.S. adults in their health practices, linguistic repertoires, and lifestyles. Nevertheless, past interventions with this population have failed to take advantage of traditional Chinese practices, such as tai-chi, the use of medicinal herbs, cupping, and qi gong (Wang & Xiong, 2013), in HTN management programs (Li, Stewart, Stotts, & Froelicher, 2005; Li, Stotts & Froelicher, 2008; Li, Wallhagen et al., 2008). Thus, a culturally- and linguistically-appropriate intervention is imperative to help Chinese adults achieve better HTN management.

A study found that use of bicultural and bilingual community health coaches (CHCs) was effective in improving diabetes management in Chinese adults (Ivey et al., 2012), but they have not been used in HTN management. There is no single clear and established definition of health coaching. A systematic review concluded that the majority of studies that implemented health coaches had the following characteristics in common: patient-centeredness, patient-determined goals, use of a self-discovery process, and accountability (Wolever et al., 2013). Research has shown that there is a lack of accountability between physicians and their patients, which shows up as poor medication adherence and BPC. Social accountability encourages patients to adhere to their medical treatment in-between physician appointments (Oussedik et al., 2017). To address these considerations, the CHC method was chosen for this study because it is characterized by frequent and periodic contact. Additionally, almost half of all health coaches were people who had experience in healthcare and who typically received 16 to 23 hours of training (Wolever et al., 2013). Studies implementing the use of CHCs in other populations, including other ethnic minority groups (e.g., individuals who only spoke Spanish), individuals and families of low socioeconomic status, and rural communities concluded that the intervention was effective for HTN management and improvement (Bennett et al., 2009; Dye, Williams, & Evatt, 2014; Thom et al., 2016; Wu et al., 2018). For example, a “teamlet” model was proposed by Bodenheimer and colleagues (Bodenheimer, 2005a, 2005b; Bodenheimer, Lorig, Holman, & Grumbach, 2002) to expand the traditional 15-minute medical visit. The authors of this study adopted the “teamlet” model but expanded the teamlet coach role to CHCs, who previously had health-related work backgrounds, were culturally and linguistically competent in taking care of Chinese immigrants, and received training focusing on HTN management (3-hour face-to-face training) aiming to improve patients’ HTN self-management. Bodenheimer and Laing’s teamlet coach met one-on-one with a patient in an individual counseling session with the goal of enhancing their healthcare experience and self-management skills (Bodenheimer & Laing, 2007). The original teamlet approach did not specifically address cultural and linguistic factors. However, a culturally sensitive HTN management intervention delivered by a culturally and linguistically competent CHC may help overcome cultural barriers to treatment for racial/ethnic minorities to improve their disease management. Research has shown that racial/ethnic minorities are usually limited in their English proficiency, health literacy, and expression of their health needs (Li et al., 2006; Li, Stotts et al., 2008; Sentell & Braun, 2012). Thus, there is a need to develop and test the efficacy of a culturally- and linguistically-sensitive HTN intervention with CHCs trained to guide Chinese adults in better managing their HTN.

## Aims and Innovation

The purpose of this study was to develop and pilot test the efficacy of a culturally- and linguistically-sensitive CHC-based intervention in Chinese immigrants to improve medication adherence and BPC. If the proposed CHC-based intervention is shown to be effective, there would be the potential that this intervention can be further refined for the broader clinical setting which aims to help Chinese immigrants improve their HTN management. The study is innovative because: (1) The CHC-based intervention was newly developed based on cultural practices of Chinese adults and specifically addresses HTN management (e.g., incorporating Chinese medicine into Western medicine); (2) the CHC-based intervention is low cost considering that the CHCs are research assistants/nursing assistants who have baseline knowledge of HTN management, so no compensation for training was required; (3) the CHC-based intervention can be adopted to other Asian immigrants who share similar cultural backgrounds with Chinese immigrants.

## Method

This study was conducted in 2017 with a one group, pre- and post-test design. Two approaches were used: (1) self-report questionnaires to collect demographic information, cultural factors, and medication adherence; and (2) measures of blood pressure. Institutional review board approval was obtained from a university in San Francisco (#X17-41).

### Setting

The setting where participants were recruited is a senior low-income housing apartment complex subsidized by the federal government Housing and Urban Development Division. It is located near Chinatown in the San Francisco Bay Area. It serves around 200 senior citizens, 80% of whom are Chinese immigrants.

### Sample

Fifty-five Chinese immigrants with HTN were approached and 23 participated and completed the study. The response rate was 41.8%. A convenience sample of 23 Chinese immigrants with HTN was recruited from the aforementioned senior housing apartment complex. Inclusion criteria were: (1) self-identified as a Chinese immigrant aged 18 years and older; (2) having a diagnosis of HTN; (3) having taken HTN medications for more than one month before study enrollment; and (4) being able to speak and read Chinese. Exclusion criteria were based on self-report: being medically unstable (e.g., acute renal failure) or having concurrent psychiatric problems (e.g., schizophrenia).

### Sample Size

The sample size (*n* = 23) was determined based on the resources available in the study period and the study objectives.

### Measurements

All questionnaires, including demographic information, clinical factors, and medication adherence were pencil and paper and completed by the study participants.

*Duration of HTN Diagnosis* (years) referred to the duration of time from first diagnosis of HTN to the study interview.

*Number of Antihypertensive Medications* was defined as the medications that the patient took regularly for more than a month.

#### Measurement of Blood Pressure

An Omron brand digital BP machine (code: HEM-7201) was used to measure patient blood pressure following the standard processes by the Joint National Committee VII (Joint National Committee, 2003). Blood pressures were obtained twice. The values for systolic and diastolic blood pressures were averaged.

#### Measurement of Medication Adherence

The *Medication Adherence Scale* contains 3 items regarding whether patients forgot, missed, or were careful about taking their medication (Li et al., 2005). The scale was modified from Morisky, Green, and Levine (1986). The scale was translated into Chinese; and the format of the questions and response choices were revised. That is, instead of yes/no, a Likert scale ranging from “None of the time” to “All of the time” to better reflect Chinese culture and Chinese preferences in answering questions (Li et al., 2005). The reliability was 0.65 in Chinese immigrants (Li et al., 2005). The scores for the three items were summed; scores ranged from 3 to 15, with 3 meaning worst adherence and 15 representing the best adherence.

## Intervention

The CHC intervention included the following components:

First, two CHCs measured participants’ BP (The training for two CHCs is described in the section of “study procedure”).

Second, one trained CHC did a 20-minute PowerPoint-based group presentation on HTN management in the initial visit. The content included general strategies for managing HTN: (1) incorporating Chinese health practices (e.g., performing Tai-Chi); (2) reduction of salt intake by replacing salt with Chinese herbs and spices; and (3) the importance of adherence to antihypertensive medications to achieve optimal BP control.

Third, a 5-minute story-telling video was shown to the entire group right after slide presentation in the initial visit. This video was developed by the first author about a Chinese woman’s experience regarding the following aspects: (1) her HTN management; (2) how she developed a stroke, secondary to uncontrolled HTN (note: stroke is reported to be the most commonly seen complication due to uncontrolled HTN in Chinese (Fang et al., 2006)); (3) how she recovered from the stroke; (4) what strategies she used to prevent a stroke from happening again due to uncontrolled HTN; and (5) her advice for her hypertensive fellows to prevent severe complications, such as stroke, by optimizing their HTN management.

Fourth, the CHC who did the presentation conducted a 10-minute question-and-answer session in a group format at the end of initial visit.

Fifth, following the initial visit, the study participants were invited for four bi-weekly group follow-up sessions, with each session consisting of a BP check and a 10-minute group question-and-answer session. All four follow-up sessions were conducted by the CHC who did the presentation in the initial visit.

The decisions about format of the delivery of our intervention, content of the sessions, number of sessions, length of each session (related to ‘dose’), and duration of the intervention were made based on the first author’s published pilot studies (Li, Gomez, & Tam, 2015; Li, Lu, & Huang, 2019).

## Study Procedures

Two bilingual and bicultural CHCs were recruited and trained before the study launch. The CHCs had over 100 hours of experience working in the hospital, including working as a nursing assistant and having experience with Chinese patients. They were trained by the PI for two days in the following areas: (1) measurement of BP according to Joint National Committee VII guideline; (2) basic information on HTN; (3) pharmacological and nonpharmacological treatment for HTN; (4) interview and counseling techniques; and (5) role playing for interviewing/counseling.

During the initial visit with the study participants, the CHCs obtained a written consent indicating agreement to participate. The participants first completed pencil-and-paper, self-reported questionnaires on the following variables:

Sociodemographic and cultural data, such as age, gender, and cultural beliefs.

Clinical data, such as smoking history.

Once the participant completed the questionnaires, the CHCs double-checked to make sure the questionnaires were fully and properly completed. Then the CHCs measured the participant’s BP twice in the sitting position using a calibrated, digital BP machine and applying standard procedures suggested by the Joint National Committee VII (Joint National Committee, 2003). The average of the two readings was computed, which served as baseline data. If the two BPs were more than 5 mmHg different, then another BP was taken and the average of the three was recorded. Afterwards, one trained CHC did a 20-minute slide presentation on HTN management to the group of 23 participants. After the presentation, the 5-minute story-telling video was presented to the entire group. After the education presentation, the same CHC who did a presentation conducted a 10-minute question-and-answer session for the group. Following the initial visit, the study participants were invited for four bi-weekly 10-minute group follow-up question-and-answer sessions, which were conducted by the CHC who did an initial presentation. At the end of each session, the participant received a small incentive item, such as a shopping bag and magnifier. In addition, at the end of the final visit, each participant received a hot/cold insulated water bottle as a final thank-you gift to acknowledge their time for taking part in the study.

### Data Analysis

All data were analyzed using IBM SPSS Statistics version 24 (IBM, Inc., Armonk, NY, USA). Descriptive statistics were used to screen data for missing values and outliers and to describe the demographic and clinical variables. A paired sample *t*-test was used to examine the difference between pre- and post-intervention in terms of change in BP and medication adherence. Statistical significance was set at 0.05.

## Results

### Sample Characteristics

Table 1 shows the demographic, cultural, and clinical data of study participants. The majority of the participants were female (65.2%), middle school educated (47.8%), married (65.2%), not religious (69.6%), employed (69.6%), and made an annual income less than $9,999 (52.2%). In terms of cultural and linguistic background, a majority of them were first-generation Chinese (82.6%), born in Mainland China (91.3%), and spoke Cantonese (69.6%). A very high percentage smoked (82.6%). Less than 10% of the participants had their blood pressure controlled (8.70%). The baseline BMI was 25.6 kg/m2.

**Table 1:** Baseline Characteristics of the Sample of Chinese Immigrants with Hypertension (n = 23)

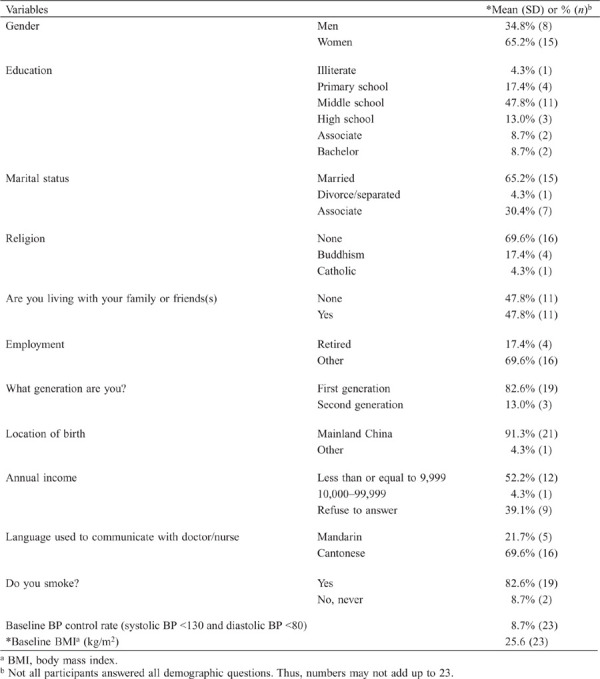

### Change in Outcomes Over a 2-Month Period

Table 2 represents the blood pressure changes over the duration of the study. The baseline systolic BP was 152.33 (±12.15) mmHg and diastolic BP was 86.50 (±11.78) mmHg. At week 8 (post-intervention), we observed a significant reduction of both systolic BP (−17.33 ± 11.32 mmHg, *p* < 0.005) and diastolic BP (−9.58 ± 6.57, *p* < 0.005) (see Table 2). The mean score for medication adherence was 10.56 (±3.24) (possible range 3–15) at baseline and there was no significant change at week 8 (10.89 ± 3.95, *p* = 0.86) (Table 2).

**Table 2:** Changes in Blood Pressure (BP) Over the 8-Week Intervention Period (mmHg)

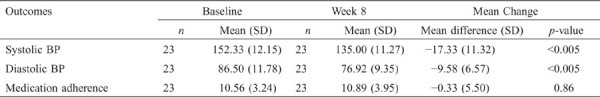

## Discussion

This study describes the development and implementation of a culturally tailored CHC intervention for self-identified Chinese immigrants with HTN to evaluate its efficacy in improving HTN management. Of the 23 participants, the majority were first-generation, Cantonese-speaking females born in Mainland China, middle-school educated, married, not religious, employed with an annual income of less than $9,999, and smokers.

The results demonstrated that a multicomponent intervention, including a 25-minute educational presentation (i.e., 20-minute presentation & 5-minute video), followed by five 10-minute bi-weekly question-and-answer sessions with the last session conducted at 2-months from baseline, resulted in significant reductions in both systolic and diastolic BP. Compared to the study conducted in the general American population which also implemented health coaches for HTN management (Dye et al., 2014), these results showed more promising efficacy (reduction in systolic BP −17.33 versus −5.78 mmHg and reduction in diastolic BP −9.58 versus −1.12 mmHg).

Different from our intervention which consisted of a group presentation and five question-and-answer sessions (all sessions are in person visits), Dye et al. (2014) offered an in-person baseline coaching session followed by eight 30-minute coaching sessions by telephone. As Dye et al. (2014) stated, some of the challenges to lifestyle behavior changes in the participants included a lack of understanding of health educational materials and a lack of social support. This may explain why our one group presentation and five in-person sessions were more efficacious than Dye et al.’s (2014) one personal visit and eight phone calls. Our intervention also showed better efficacy compared to Wu et al. (2018) (reduction in systolic BP −17.33 versus −5.90 mmHg and reduction in diastolic BP −9.58 versus −5.50 mmHg). Similar to Dye et al.’s (2014) study, Wu et al. (2018) provided 12 monthly coaching phone calls, none of which was a personal visit, which may explain why their intervention had less efficacy than our culturally sensitive, CHC-based intervention with five in-person visits. Additionally, our study recruited participants who lived in urban areas—which provide more convenient access to medical care—as opposed to the participants in Wu et al.’s (2018) who lived in rural areas. Given this, replication of our study should be implemented in rural areas and with participants who have similar baseline HTN levels to compare the results.

In terms of medication adherence, the CHC intervention did not show significant results in our study (i.e., nonsignificant improvement in medication adherence). One study involving HTN management showed that in general, American patients with HTN who were coached showed improvement in their medication adherence compared to those who were not coached after a 12-month follow-up (Thom et al., 2015). There are a few possible reasons for the discrepancies between our findings with Thom et al.’s (2015). First, we had a smaller sample size (*n* = 23 versus *n* = 224 in Thom et al. (2015)) which may not generate sufficient data for us to fully assess medication adherence behaviors. It is suggested in the future, a larger sample be recruited to generate more robust findings. Second, Chinese immigrants tend to over-report their medication adherence, so they do not disappoint their healthcare providers. Maintaining a harmonious relationship by pleasing the authorities and over-reporting (correcting) their negative health behaviors is an important Asian cultural value (Lihua, 2013). This may be an important cultural difference between Chinese immigrants and the general population which deserves more investigation to improve medication adherence in the Chinese immigrant population. For instance, in Thom et al.’s (2015) study, the majority of the participants were Latino/Hispanic followed by African Americans; Latinos/Hispanics and African Americans were found to have lower rates of medication adherence compared to Chinese immigrants (Tong, Chu, Fang, Wall, & Ayala, 2016). Third, medication adherence data were gathered from self-reported questionnaires whereas Thom et al. (2015) also used objective pharmacy records for comparison to what the participants reported. Given that over-reporting/correcting risk behaviors to please their healthcare providers puts patients at high risk for poor management of their chronic disease which in turn leads to further severe complications, exploration of reasons behind over-reporting of health behaviors and resolutions to correct this tendency warrants further investigation in future studies. Furthermore, using an objective measure of medication adherence in the study, such as a pharmacy record, may potentially provide a better estimate of medication adherence behaviors.

## Limitations

The study limitations included pre- and post-test design with only one arm (group), small sample size, and potentially inaccurate evaluation of exclusion criteria, such as schizophrenia. Threats to the internal validity of our one-group pre- and post-test design include not knowing for certain whether the participants’ interactions with the CHC were the sole reason for their improvement in BP. For example, it is not known if regular interaction with CHCs may have inspired participants to adopt other significant healthy lifestyle changes in addition to the education received during the study. Another explanation for the significant improvement in BP may have been due to them feeling more relaxed with the periodic BP checks over time as they became familiar with the procedure. Thus, future tests of efficacy of the proposed CHC intervention in a randomized controlled trial study with a larger sample size is highly recommended to establish a robust intervention program.

In the future, expanding our research to Chinese immigrant communities across different states and cities is needed to recruit a larger population of Chinese immigrants. However, even with the small sample size, we were able to demonstrate potential efficacy of the proposed CHC-based HTN management program. It is also suggested that a similar intervention program be replicated in future studies using a design of a randomized clinical trial with two arms (intervention versus control), a longer study period, and objective medication adherence measures to generate more robust results.

There is the possibility that we did not accurately exclude patients who were being medically unstable (e.g., acute renal failure) or having concurrent psychiatric problems (e.g., schizophrenia) as those exclusions were based on self-report. However, we trained our CHCs to not only ask patients self-report those ineligible conditions but also to identify potential signs of medical or mental discomforts which could have prevented participants from providing reliable data.

## Summary

Our CHC-based HTN management program showed promising outcomes in helping Chinese immigrants manage their HTN in terms of reduction of both systolic and diastolic BPs. Although the intervention did not improve medication adherence in our study, it may show the efficacy in adherence if an objective measure of medication adherence is used as Chinese immigrants tend to over-report their adherence. Nevertheless, given the efficacy in reducing blood pressures, potentially low-cost and ease of implementation, a CHC-based HTN management program is recommended to help Chinese immigrants achieve optimal HTN management.

## Implications for Clinical Practice

Since a CHC-based intervention may be economical and easily implemented, once it is determined to be effective with a larger sample of Chinese immigrants in a more rigorous randomized controlled trial, this can be disseminated and implemented in a wide range of clinical settings which serve Chinese immigrants who need more intensive HTN management. Given a very low BP control rate in Chinese immigrants in the United States, a cost-effective and efficacious HTN management program is essential to help this population optimize their BPC. Furthermore, a similar intervention program may be applicable to other chronic diseases which share similar features of HTN, such as diabetes.

It is noteworthy that such a management program usually requires the researcher to be familiar with the health needs and cultural backgrounds of the target populations in order to find a suitable, reliable, and trusted CHC to facilitate a HTN management program. Thus, a researcher should have been working within the Chinese community to gain trust from this population. In addition, a CHC should be culturally and linguistically competent in order to generate the best results in HTN management. Our study showed that a CHC located in the target community is an efficient approach for Chinese immigrants.

## Acknowledgements

The authors wish to acknowledge Chinese immigrants who participated in this study.

## Declaration of Conflicting Interests

The authors declared no potential conflicts of interest concerning the research, authorship, or publication of this article.

## Funding

This project was supported by the American Heart Association Community Grant and San Francisco State University, Development of Research and Creativity (DRC) Grants.
